# Inter-observer and segmentation method variability of textural analysis in pre-therapeutic FDG PET/CT in head and neck cancer

**DOI:** 10.1371/journal.pone.0214299

**Published:** 2019-03-28

**Authors:** Catherine Guezennec, David Bourhis, Fanny Orlhac, Philippe Robin, Jean-Baptiste Corre, Olivier Delcroix, Yves Gobel, Ulrike Schick, Pierre-Yves Salaün, Ronan Abgral

**Affiliations:** 1 Department of Nuclear Medicine, Brest University Hospital, Brest, France; 2 Imagerie Moléculaire in Vivo, CEA-SHJF, Inserm, CNRS, Université Paris-Sud, Université Paris-Saclay, Orsay, France; 3 Department of Head and Neck Surgery, Brest University Hospital, Brest, France; 4 Department of Radiotherapy, Brest University Hospital, Brest, France; Roswell Park Cancer Institute, UNITED STATES

## Abstract

**Aim:**

Characterizing tumor heterogeneity with textural indices extracted from 18F-fluorodeoxyglucose positron emission tomography (FDG PET/CT) is of growing interest in oncology. Several series showed promising results to predict survival in patients with head and neck squamous cell carcinoma (HNSCC), analyzing various tumor segmentation methods and textural indices. This preliminary study aimed at assessing the inter-observer and inter-segmentation method variability of textural indices in HNSCC pre-therapeutic FDG PET/CT.

**Materials and methods:**

Consecutive patients with HNSCC referred in our department for a pre-therapeutic FDG PET/CT from January to March 2016 were retrospectively included. Two nuclear medicine physicians separately segmented all tumors using 3 different segmentation methods: a relative standardized uptake value (SUV) threshold (40%SUVmax), a signal-to-noise adaptive SUV threshold (DAISNE) and an image gradient-based method (PET-EDGE). SUV and metabolic tumor volume were recorded. Thirty-one textural indices were calculated using LIFEx software (www.lifexsoft.org). After correlation analysis, selected indices’ inter-segmentation method and inter-observer variability were calculated.

**Results:**

Forty-three patients (mean age 63.8±9.3y) were analyzed. Due to a too small segmented tumor volume of interest, textural analysis could not be performed in 6, 11 and 15 cases with respectively DAISNE, 40%SUVmax and PET-EDGE segmentation methods. Five independent textural indices were selected (Homogeneity, Correlation, Entropy, Busyness and LZLGE). There was a high inter-contouring method variability for Homogeneity, Correlation, Entropy and LZLGE (p<0.0001 for each index). The inter-observer reproducibility analysis revealed an excellent agreement for 3 indices (Homogeneity, Correlation and Entropy) with an intraclass correlation coefficient higher than 0.90 for the 3 methods.

**Conclusions:**

This preliminary study showed a high variability of 4 out of 5 textural indices (Homogeneity, Correlation, Entropy and LZLGE) extracted from pre-therapeutic FDG PET/CT in HNSCC using 3 different contouring methods. However, for each method, there was an excellent agreement between observers for 3 of these textural indices (Homogeneity, Correlation and Entropy).

## Introduction

Solid malignancies usually show high levels of biologic heterogeneity, in terms of hypoxic and necrotic regions, variability in cellular proliferation and intra-tumoral angiogenesis. Taking this heterogeneity into account could help improve patients’ therapeutic management, classifying patients between different risk subgroups [1][2][3].

This could be particularly useful in head and neck squamous cell carcinoma (HNSCC) which typically presents a high biologic heterogeneity [4][5][6]. HNSCC is the sixth most common malignancy by incidence worldwide and includes cancers of the oral cavity, oropharynx, hypopharynx and larynx [7]. Therapeutic strategies rely on usual prognostic factors, such as the tumour size, nodal involvement and distant metastasis [8][9], the anatomic subsite, or the human papilloma virus (HPV 16, 18) infection status [10]. Despite aggressive treatment strategies, it presents a high rate of loco-regional recurrence (up to 40%) [11]. Thus predicting tumor response to therapy remains difficult and could benefit from heterogeneity analysis.

Pre-therapeutic 18F-Fluorodeoxyglucose positron-emission tomography (FDG PET/CT) is recommended in locally advanced HNSCC to assess remote extension and in order to search for synchronous cancer [12]. Several studies already suggested its prognostic significance in selecting patients at risk of recurrence using different standard quantitative parameters, such as static (SUV = Standardized Uptake Value) or volumetric (MTV = metabolic tumor volume; TLG = total lesion glycolysis) parameters [13][14][15]. Some tools have recently been developed to allow radiomics extraction of PET image-derived heterogeneity biomarkers [16]. Therefore, besides standard PET quantitative parameters, textural indices could also have a prognostic value.

There are some challenges to handle with textural analysis. Indeed, various parameters have to be settled when calculating textural indices, such as the segmentation method to delineate a tumor volume of interest, the resampling method, matrix definitions and indices formula. The robustness of these indices towards those parameters should be evaluated [17]. The correlation of textural indices between themselves and with standard PET quantitative parameters should also be considered, since some indices have very similar definitions [18].

Several studies have showed promising results to predict survival in patients with HNSCC, analyzing various cancer anatomic subsites, tumor segmentation methods and textural indices [19][20][21]. However, as in other solid cancers, no consensus has been reached in HNSCC on the best segmentation method to use and on the most adapted textural indices to study.

The objective of this preliminary study was to assess the reproducibility between 2 observers and the variability between 3 contouring methods when calculating different textural indices on HNSCC pre-therapeutic FDG PET/CT.

## Materials and methods

The institutional ethics committee of Brest University hospital (Number 2017.CE25) approved this study and all patient signed a written consent form.

### Patients

Consecutive patients referred for FDG PET/CT for the assessment of a Head and Neck cancer to the nuclear medicine department of Brest University Hospital (France) were retrospectively analysed from January 2016 to March 2016. Inclusion criteria were a pre-treatment FDG PET/CT (at initial staging) and a biopsy proven HNSCC. Patients were excluded if they were less than 18 years old or if they had a history of head and neck malignancy.

### Image acquisition

FDG PET/CT images were acquired on two Biograph-mCT systems (Siemens, Erlangen, Germany) with the same technical features. Patients were required to fast at least 6 hours before injection so that their serum glucose level would be appropriately low (<7mmol/L for non-diabetic patients and <9mmol/L for diabetic patients). Images were performed 60 minutes after injection of approximately 3 MBq/kg of FDG (IBA Molecular Imaging, Saclay, France).

CT scan was obtained first in the craniocaudal direction using a whole-body protocol, 55 seconds after injection of intravenous iodine contrast agent (1.5 mL/kg), without any bolus tracking, unless contraindicated. CT consisted in a 64-slice multidetector-row spiral scanner with the following standard parameters: transverse field of view = 700 mm, collimation = 16 x 1.2 mm, pitch = 1, automatic tube potential modulation (carekV), and automatic tube current modulation (care4D).

PET images were acquired in the craniocaudal direction using a whole-body protocol (2 minutes per step) and were reconstructed using an ordered subset expectation maximization (OSEM) algorithm (True X = point spread function + time of flight compensation ordered subset expectation maximization-3D). The images were corrected for random coincidences, scatter, and attenuation using the CT scan data. PET images were smoothed with a Gaussian filter (full-width at half-maximum = 2 mm). The reconstruction transaxial matrix size was 200 x 200 voxels with voxel size = 4.07 x 4.07 x 2 mm.

### Images analysis

All primary tumors were segmented using 3 different contouring methods by two nuclear medicine physicians with oncology expertise, independently, creating volumes of interest (VOI). The 3 different delineation methods consisted in a fixed SUV threshold method, containing voxels equal or greater than 40% of SUV maximum value (40%SUVmax) [22][23], a signal-to-noise adaptative SUV threshold method (DAISNE) [24], and an image gradient-based method (PET-EDGE) [25] using MIM software (MIM Software Inc., Cleveland, United-States).

No spatial discretization was used. Texture analysis was performed after applying an absolute resampling method to voxels intensities with 64 discrete values and bounds set to 0 and 30 SUV, corresponding to the typical range of tumor SUVs encountered in HNSCC [26].

In each VOI, standard PET quantitative parameters were measured (SUVmax, MTV) and thirty-one textural indices were calculated using LIFEx software (www.lifexsoft.org) [16] (Table 1). Theses indices were extracted from 4 different matrices which were computed for each VOI: the Gray-Level Cooccurence Matrix (GLCM), the Gray-Level Run Length Matrix, the Neighborhood Gray-Level Dependence Matrix (NGLDM) and the Gray-Level Zone Length Matrix (GLZLM). GLCM characterizes how often pairs of voxels with specific SUV values can be found at a specific distance in a specific direction and was computed using a distance of 1 voxel and 13 directions [27]. GLRLM gives the size of homogeneous runs for each gray-level and was computed using 13 directions [28]. NGLDM corresponds to the difference of gray-level between one voxel and its 26 neighbours in 3 directions [29]. GLZLM gives the size of homogeneous zones for each gray-level in 3 dimensions [30]. Textural indices could only be computed by the software for VOI equal or greater than 64 voxels, corresponding to a minimum volume of 2.12ml (voxel size of 4.07x4.07x2mm) and containing only one cluster. For VOI containing more than one cluster, the most representative one was manually selected by the operator based on its uptake intensity and volume.

**Table 1:** Textural indices.

Matrix	Index
Gray-Level Cooccurence Matrix (GLCM)	Homogeneity, Energy, Contrast_glcm, Correlation, Entropy, Dissimilarity
Gray-Level Run Length Matrix (GLRLM)	SRE (Short-Run Emphasis), LRE (Long-Run Emphasis), LGRE (Low Gray-Level Run Emphasis), HGRE (Hign Gray-Level Run Emphasis), SRLGE (Short-Run Low Gray-Level Emphasis), SRHGE (Short-Run High Gray-Level Emphasis), LRLGE (Long-Run Low Gray-Level Emphasis), LRHGE (Long-Run High Gray-Level Emphasis), GLNUr (Gray-Level Non Uniformity for run), RLNU (Run Length Non Uniformity), RP (Run Percentage)
Neighborhood Gray-Level Dependence Matrix (NGLDM)	Coarseness, Contrast, Busyness
Gray-Level Zone Length Matrix (GLZLM)	SZE (Short-Zone Emphasis), LZE (Long-Zone Emphasis), LGZE (Low Gray-Level Zone Emphasis), HGZE (High Gray-Level Zone Emphasis), SZLGE (Short-Zone Low Gray-Level Emphasis), SZHGE (Short-Zone High Gray-Level Emphasis), LZLGE (Long-Zone Low Gray-Level Emphasis), LZHGE (Long-Zone High Gray-Level Emphasis), GLNUz (Gray-Level Non Uniformity for zone), ZLNU (Zone Length Non Uniformity), ZP (Zone Percentage)

### Statistical analysis

Correlations between textural indices and standard PET quantitative parameters were estimated using Pearson coefficients. Pairs of features with a Pearson correlation coefficient higher than 0.8 were considered very highly correlated. Groups of highly correlated parameters were extracted with the same method as Orlhac et al. [18]. One parameter from each independent group was selected for analysis. Bland Altman plots were used to compare VOI between segmentation methods. Indices variability between contouring methods was analysed for each selected textural index with Friedman and Wilcoxon tests. Inter-observer reproducibility was assessed using an intraclass correlation test. Based on Landis and Koch scale, agreement between operators was considered excellent if the intraclass correlation coefficient (ICC) was superior to 0.8, great for ICC between 0.61 and 0.8, moderate for ICC between 0.41 and 0.6, low for ICC between 0.21 and 0.4 and poor for ICC ≤ 0.2. Significance level of p-value was 0.05. Statistics were realized with XLStat software (Addinsoft, Paris, France).

## Results

### Patients

Between January and March 2016, 43 patients were included. All patients had a whole-body FDG PET/CT after injection of 3.09 ± 0.14 MBq/kg of FDG. Glucose level prior to the acquisition was 6.4 ± 1 mmol/L and 29 patients (67%) received iodine contrast agent. Due to a too small segmented tumor VOI (<2.12ml), textural analysis could not be performed in 6, 11 and 15 cases with respectively DAISNE, 40%SUVmax and PET-EDGE segmentation methods. All patients with a too small VOI with either DAISNE or 40%SUVmax also had a too small VOI with PET-EDGE method so that 28 patients (male = 24, female = 4, mean age ± SD = 64.8 ± 9.8 years) had large enough segmented VOI with the 3 segmentation methods to allow all features’ calculation (31 textural indices, SUVmax and MTV). Patients’ characteristics are shown in Table 2.

**Table 2:** Characteristics of patients.

Characteristics		Patients (n = 28)
Age, y, mean ± SD		64.8 ± 9.8
Sex, M/F		24/4
Tumor location, no. of patients (%)		
	Oral cavity	8 (28)
	Oropharynx	10 (36)
	Hypopharynx	6 (21)
	Larynx	1 (4)
	Extended (≥ 2 subsites)	3 (11)
AJCC stage, no. of patients (%)		
	I	0 (0)
	II	3 (11)
	III	2 (7)
	IV	23 (82)
T classification, no. of patients (%)		
	T1	0 (0)
	T2	7 (25)
	T3	5 (18)
	T4	16 (57)

### Correlation analysis

After correlation analysis of each of the 31 textural indices with all the other indices, 9 groups of highly correlated parameters were extracted. Nine independent textural indices were selected: Homogeneity, Correlation, Entropy, Busyness, LZLGE, LZHGE, LGZE, HGZE and GLNUz (Table 3). After correlation analysis of these 9 textural indices with standard PET quantitative parameters, LGZE and HGZE were significantly correlated with SUVmax (r = -0.89, p < 0.0001 and r = 0.97, p < 0.0001 respectively) while GLNUz and LZHGE were significantly correlated with MTV (r = 0.96, p < 0.0001 and r = 0.84, p < 0.0001). Five textural indices were finally selected: Homogeneity, Correlation, Entropy, Busyness and LZLGE. Correlation coefficients are shown in Table 4.

**Table 3:** Groups of highly correlated indices.

Groups of highly correlated indices	Absolute correlation coefficients mean ± SD
Homogeneity, Contrast_glcm, Dissimilarity, SRE, LRE, RP, Contrast, SZE, ZP	0.89 ± 0.08
LGZE, SZLGE, LGRE, SRLGE, LRLGE, Energy	0.93 ± 0.08
HGZE, SZHGE, HGRE, SRHGE, LRHGE	0.99 ± 0.01
GLNUz, GLNUr, RLNU	0.94 ± 0.03
Entropy, Coarseness, ZLNU	0.78 ± 0.08
LZHGE, LZE	0.85
Correlation	-
Busyness	-
LZLGE	-

**Table 4:** Textural indices correlation coefficient between themselves and with PET standard quantitative parameters (Pearson test).

Parameters	SUVmax	MTV	Homogeneity	Correlation	Entropy	Busyness	LZLGE
SUVmax	1	-0.25	-0.69	-0.22	0.33	0.23	-0.48
MTV		1	0.69	0.69	0.55	0.17	0.73
Homogeneity			1	0.67	0.24	-0.15	0.69
Correlation				1	0.52	0.07	0.58
Entropy					1	0.21	0.07
Busyness						1	0.03
LZLGE							1

### Inter-contouring method reproducibility

**Fig 1:**
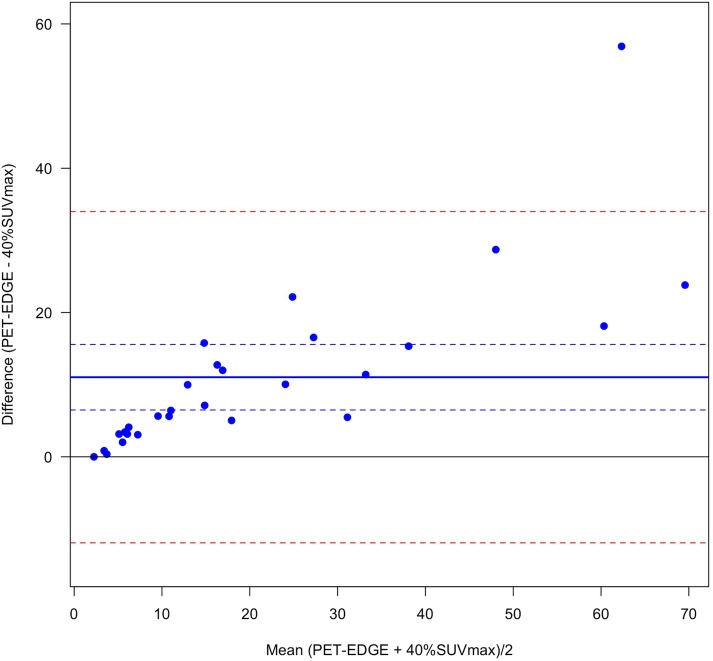
Variability between PET-EDGE and 40%SUVmax method. Bland-Altman plot. (Solid blue line) Bias. (Dashed blue lines) Bias 95% confidence interval. (Dashed red lines) Difference 95% confidence interval.

**Fig 2:**
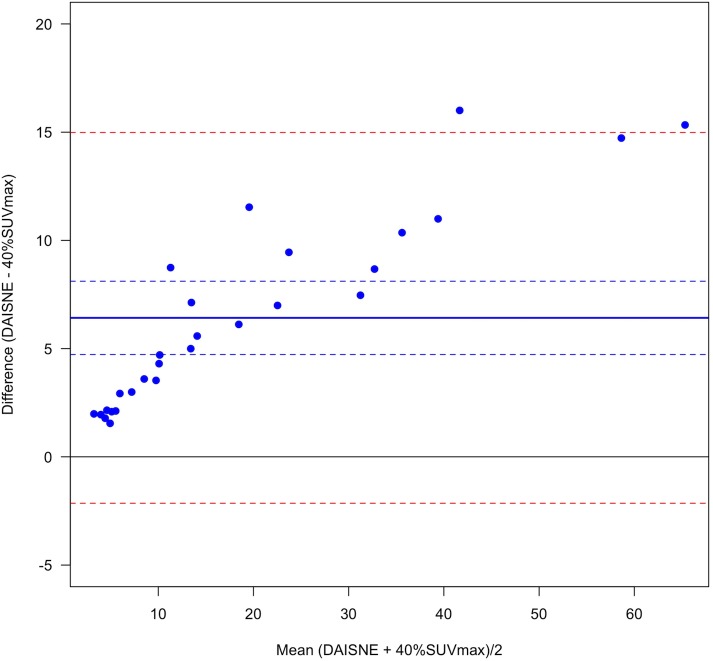
Variability between DAISNE and 40%SUVmax method. Bland-Altman plot. (Solid blue line) Bias. (Dashed blue lines) Bias 95% confidence interval. (Dashed red lines) Difference 95% confidence interval.

**Fig 3:**
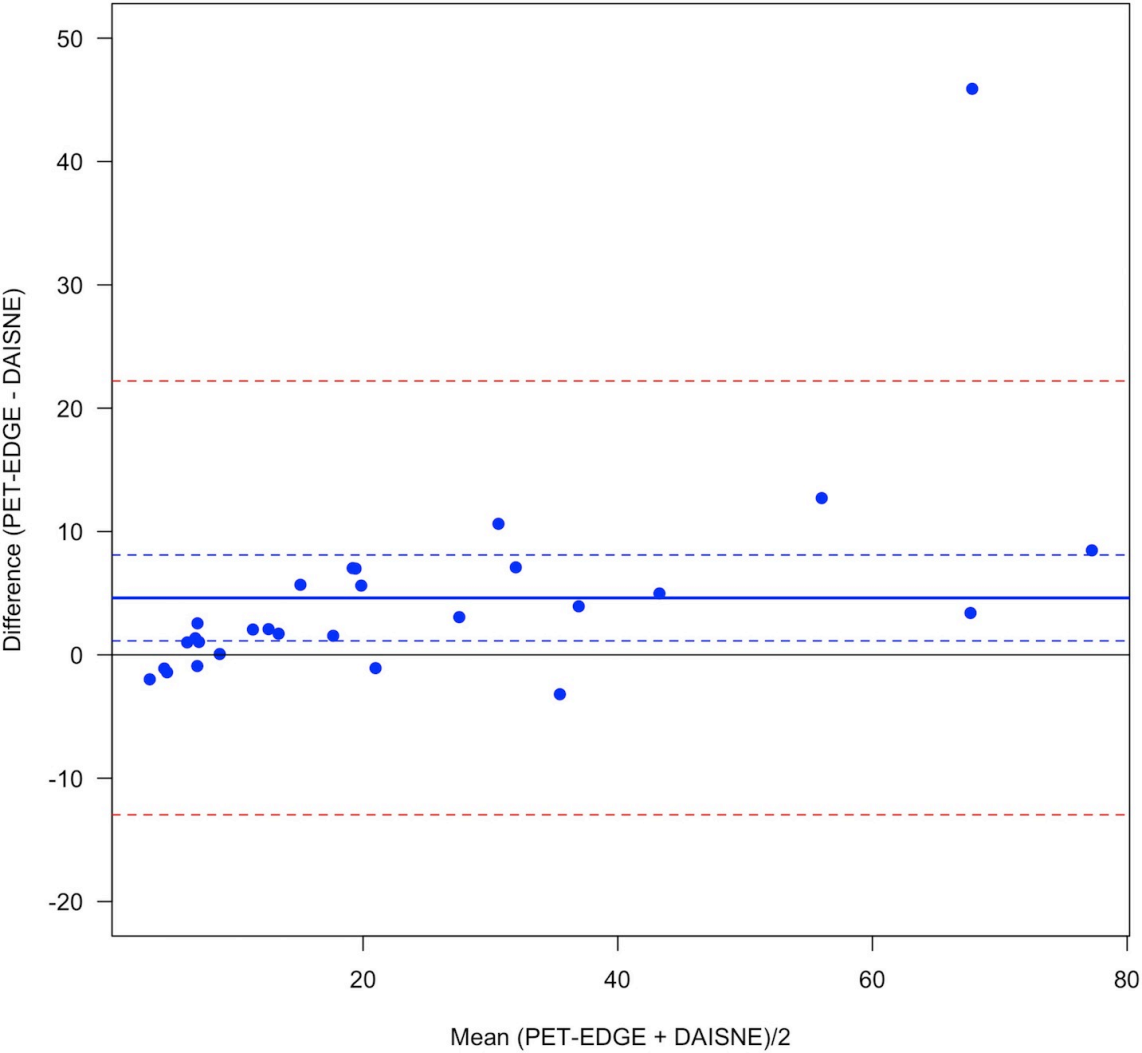
Variability between PET-EDGE and DAISNE method. Bland-Altman plot. (Solid blue line) Bias. (Dashed blue lines) Bias 95% confidence interval. (Dashed red lines) Difference 95% confidence interval.

**Fig 4:**
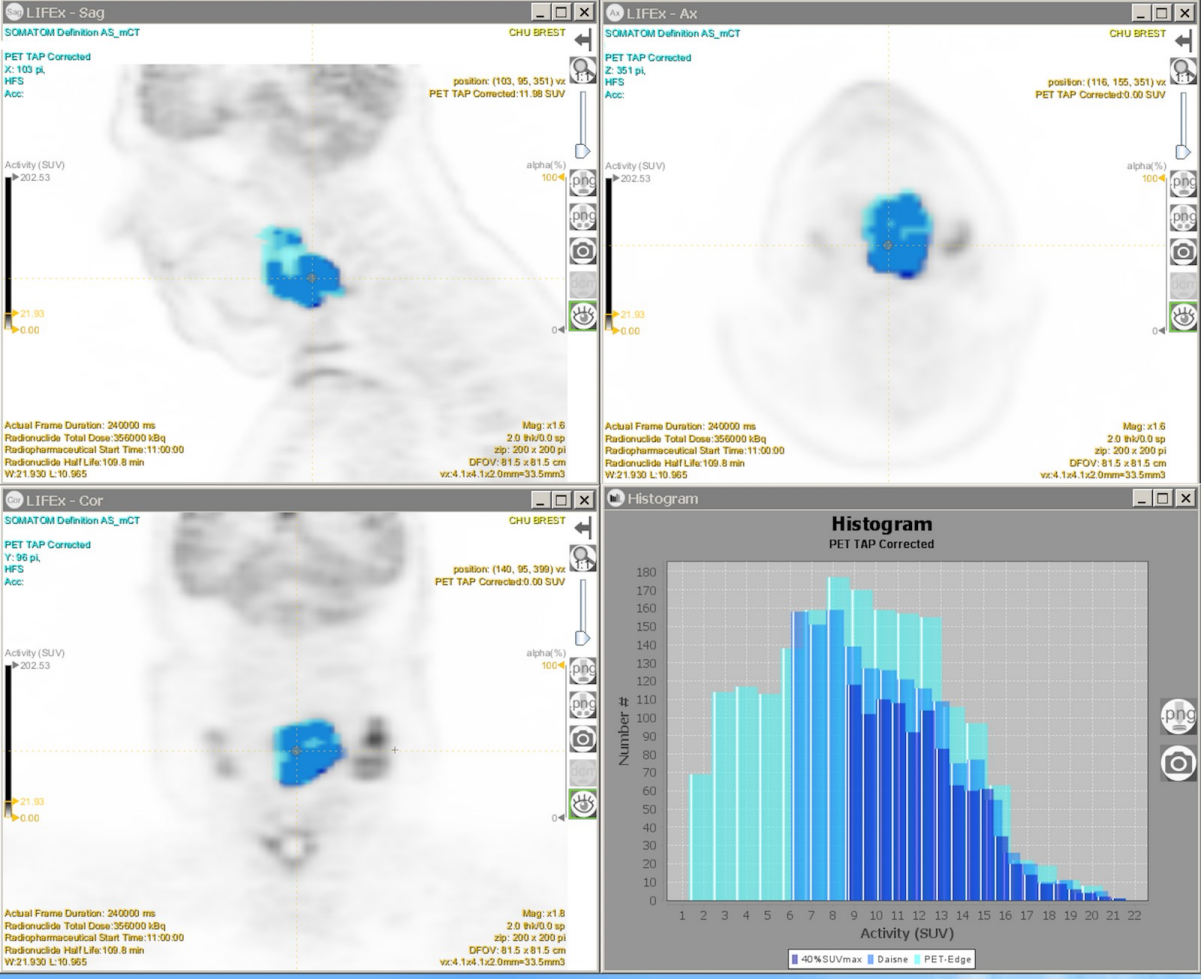
Example of VOI delineating a tumor with the 3 segmentation methods. (Turquoise blue) PET-EDGE segmentation method. (Sky blue) DAISNE segmentation method. (Dark blue) 40%SUVmax segmentation method. (Top left) FDG PET sagittal slice. (Top right) FDG PET transverse slice. (Bottom left) FDG PET frontal slice. (Bottom right) SUV histograms. With LIFEx software.

VOI were significantly different between the 3 segmentation methods. VOI were always higher with PET-EDGE or DAISNE when compared to 40%SUVmax (bias = 11.0 ± 11.7 and bias = 6.4 ±, 4.4 respectively for PET-EDGE versus 40%SUVmax and DAISNE versus 40%SUVmax) (Figs 1 and 2). The difference was less pronounced between PET-EDGE and DAISNE methods (bias = 4.6 ± 9.0) (Fig 3). An example depicting the VOI segmented with the 3 methods is shown in Fig 4.

Four of the 5 textural indices, Homogeneity, Correlation, Entropy and LZLGE, were significantly different between the 3 contouring methods (p < 0.0001 for each index). Busyness was not significantly different between the 3 contouring methods (p = 0.52) (Table 5).

**Table 5:** Inter-segmentation method variability (Friedman and Wilcoxon test p-values).

	Friedman test p-value	Wilcoxon test p-value		
Parameters	40%SUVmax vs PET-EDGE vs DAISNE	40%SUVmax vs PET-EDGE	40%SUVmax vs DAISNE	PET-EDGE vs DAISNE
SUVmax	1	1	1	1
Volume (mL)	p<0.0001	p<0.0001	p<0.0001	0.0004
Homogeneity	p<0.0001	0.0003	p<0.0001	0.20
Correlation	p<0.0001	p<0.0001	p<0.0001	p<0.0001
Entropy	p<0.0001	p<0.0001	p<0.0001	0.0002
Busyness	0.52	0.52	0.43	0.78
LZLGE	p<0.0001	p<0.0001	0.016	p<0.0001

The same results were found when comparing separately 40%SUVmax with PET-EDGE and 40%SUVmax with DAISNE. Concerning PET-EDGE with DAISNE comparison, Homogeneity was not significantly different between the 2 contouring methods; the results remained the same for the 4 other indices (Table 5).

### Inter-observer reproducibility

Homogeneity, Entropy and Correlation had an excellent inter-observer reproducibility for the 3 contouring methods with an intraclass correlation coefficient higher than 0.92, 0.90, 0.98 when considering respectively 40%SUVmax, PET-EDGE and DAISNE methods. LZLGE inter-observer reproducibility was excellent when using 40%SUVmax and DAISNE (ICC = 0.99 for both methods) and poor when using PET-EDGE (ICC = 0.04). Busyness inter-observer reproducibility was poor with the 3 methods (ICC = 0.10, ICC = -0.09 and ICC = -0.003, with respectively 40%SUVmax, PET-EDGE and DAISNE) (Table 6).

**Table 6:** Inter-observer reproducibility (intra-class correlation coefficient).

Parameters	40%SUVmax ICC	PET-EDGE ICC	DAISNE ICC
SUVmax	1	1	1
MTV	0.99	0.88	0.99
Homogeneity	0.99	0.95	0.99
Correlation	0.92	0.90	0.99
Entropy	0.99	0.92	0.98
Busyness	0.10	-0.09	-0.003
LZLGE	0.99	0.04	0.99

## Discussion

Evaluating intra-tumoral heterogeneity with textural indices may help thoroughly classifying patients between different risk subgroups. It would be particularly useful in HNSCC, conjointly with usual prognostic factors, to select patients with poor prognosis who would require intensified therapy [31].

The calculation of textural indices depends on the definition of several parameters. These parameters include the contouring method and the resampling method. Before analysing the prognostic significance of textural indices, their variability with regard to these parameters needs to be assessed. Indeed in HNSCC, no consensus has been reached on the most appropriate indices and parameters to use. The correlation between textural indices and with standard PET quantitative parameters should also be taken into account [26]. In our study, to limit the variability to these quantitative parameters, we chose to focus on only one type of tumors, HNSCC. Then, after correlation analysis, we evaluated the variability of textural indices between three segmentation methods and their reproducibility between two observers.

We chose to evaluate 3 segmentation methods, 40%SUVmax, DAISNE and PET-EDGE to contour tumors. VOI segmented with 40%SUVmax were always lower than VOI segmented with PET-EDGE and DAISNE methods, all the more since the tumor lesion was larger. Fixed threshold methods such as 40%SUVmax tend to underestimate the tumor volume and therefore may not capture regions of extremely low uptake such as necrotic regions. However a fixed threshold method may also prevent the selection of non-tumor regions close to the tumor. For textural analysis purpose, a more anatomical segmentation method, such as manual segmentation on CT, could be more appropriate to include the heterogeneity of the tumor microenvironment. Yet such a method would be less reproducible and much more time-consuming. Moreover, we retrospectively calculated the signal to background ratio (SBR). In this series, SBR was 23.5 ± 7.4 confirming that tumors were very well distinguishable from the background.

In our study, no spatial discretization was needed since all the images were acquired with exactly the same technical features, including the voxel size. We chose an absolute resampling method to discretize voxels intensities because it was shown to be more appropriate for inter and intra-patients comparison [32] and to allow both more intuitive indices’ variations to be observed and better tumor discrimination [26].

Our results showed that most textural indices extracted from pre-therapeutic FDG PET/CT were very highly correlated between themselves and with standard PET quantitative parameters. After correlation analysis of 33 parameters (31 textural indices, SUVmax and MTV), we identified 5 independent textural indices: Homogeneity, Entropy, Correlation, Busyness and LZLGE. This high correlation between textural parameters and standard PET quantitative parameters was in agreement with previous studies [18]. The high correlation between textural parameters is partly due to their definition. For example GLRLM and GLZLM are constructed based on the same principle taking into account either identical voxels in a given axis (GLRLM) and calculated an average over every axes or identical voxels in a given volume (GLZLM) [28][30]. The indices extracted from both matrices have identical definitions and therefore a high probability of being highly correlated. Since textural analysis consists in analysing the spatial distribution of voxels intensities in a given volume, there is also a possible correlation of these indices with SUVmax and MTV.

Orlhac et al. showed a high correlation within textural indices and with standard PET quantitative parameters in patients with metastatic colorectal cancers, small cell lung cancers and breast cancers [18]. They selected 6 independent textural indices (Homogeneity, Entropy, SRE, LRE, LGZE and HGZE). Using the same methodology, we also constructed subgroups of highly correlated parameters and selected 5 independent textural indices of which 2 were identical, Homogeneity and Entropy. We found a high correlation between SRE, LRE and Homogeneity, and between LGZE, HGZE and SUVmax. These results may be explained by the chosen resampling method [26].

We studied the variability of textural indices between 3 contouring methods, a fixed threshold method (40% of SUVmax), an adaptive threshold method (Daisne) and a method based on gradients (PET-EDGE). Indeed in PET characterisation of HSNCC, no contouring method has reached consensus yet [14][33]. In our series, among the 5 selected textural indices, 4 (Homogeneity, Correlation, Entropy and LZLGE) showed a high inter-contouring method variability, with a significant difference (p<0.0001). Only one index, Busyness, was reproducible between contouring methods (p = 0.52). Previous studies concerning other types of solid cancer showed various behaviours of textural indices with regard to contouring methods, some of them varying greatly. In locally advanced oesophageal cancers, comparing a fixed threshold (42% of SUVmax) with an adaptive threshold method (FLAB), Entropy showed a low to intermediate variability and Homogeneity an intermediate to very high variability [34]. In metastatic colorectal, small cell lung and breast cancers, comparing a fixed threshold (40% of SUVmax) with an adaptive threshold method (Nestle), Entropy was reproducible, Homogeneity varied moderately and Correlation, Busyness and LZLGE varied a lot between the methods [35][18]. In our study, we found a high variability of 4 out of the 5 selected textural indices, Homogeneity, Correlation, Entropy, and LZLGE, whereas Busyness was reproducible between the 3 contouring methods studied. The divergent result for Entropy could be explained by the chosen resampling method [26]. Regarding Busyness, our different result may be explained by its orders of magnitude, varying between 10^12^ and 10^15^.

We found an excellent inter-observer reproducibility for 3 selected indices (Homogeneity, Correlation and Entropy) with the 3 contouring methods (ICC>0.9), and for LZLGE (ICC = 0.99) with only 2 contouring methods (40%SUVmax and DAISNE). On the contrary, Busyness was poorly reproducible (-0.09<ICC<0.1) regardless of the contouring method. This result could also be explained by its orders of magnitude. Selecting a robust textural index in terms of inter-observer reproducibility is fundamental before assessing its prognostic value in further studies.

There are several limitations to our study.

Firstly, our study concerned a small number of patients. Indeed, this was a preliminary study to evaluate the robustness of textural indices extracted from FDG PET/CT in HSNCC. Our results should be confirmed on larger cohorts of patients.

Secondly, small tumours were an issue in textural analysis as showed in previous studies [36]. Textural indices could not be calculated in 11 (25%), 15 (34%), and 6 (14%) patients with respectively 40%SUVmax, PET-EDGE and DAISNE contouring method, because of too small delineated VOI. Textural analysis was performed with the 3 methods by the 2 observers in 28 patients (65%). The required minimum volume for analysis was 2.12ml in our study. This is consistent with previous reports focusing on several types of cancers that also fixed a minimum volume, varying from 3 to 5ml [37][36][38], thus only slightly higher than ours. Increasing the number of voxels contained in a VOI would require the acquisition of high definition images, but this would be more time-consuming and difficult to use in routine.

Thirdly, delineated contours could consist of more than one cluster, when using fixed or adaptive threshold contouring methods. Since texture calculation requires a closed delineated contour, with only one cluster, a manual intervention was then needed, either to close the contour with topologic operators or to select the most representative cluster considering its uptake intensity and volume. Thus, with the most heterogeneous tumours, the risk was greater to obtain more than one cluster, resulting in a loss of this heterogeneity information by having to adjust manually the VOI [26]. Gradient-based methods could therefore be preferred in so far as they result in a single contour. Manual segmentation on CT would also eliminate the problems of clusters. However, it would be less reproducible between observers and much more time-consuming. In our study, the inter-observer reproducibility was excellent for 3 out of the 5 selected indices with the 3 contouring methods and the reproducibility was always inferior with PET-EDGE when compared with 40%SUVmax and DAISNE.

Another limitation in our study was the possible influence of dental artifacts on textural analysis. Indeed dental artifacts or hardware were present and in the field of view of the tumor in 8 out of 43 patients (18%). Further studies would need to evaluate this influence specifically.

Finally, in this study, a Gaussian smoothing filter was applied in post-processing. Such post-processing may alter the results of textural analysis. Nevertheless, we wanted to use the same post-processing generally applied in clinical routine by the majority of centres. However, our results would have to be confirmed with other acquisition protocols and reconstruction tools.

## Conclusion

This preliminary study showed a high variability of 4 out of 5 textural indices (Homogeneity, Correlation, Entropy and LZLGE) extracted from pre-therapeutic FDG PET/CT in HNSCC between 3 contouring methods (40%SUVmax, DAISNE, PET-EDGE). However there was an excellent agreement between observers in calculating Homogeneity, Correlation and Entropy indices.

Before integrating texture analysis in overall risk stratification in HNSCC, a consensus should be reached stating which parameters to choose for calculations, particularly the segmentation method to apply. Meanwhile, comparing studies with different segmentation methods will remain difficult.
